# The daily relations of co-rumination and perseverative cognition

**DOI:** 10.1038/s41598-025-87335-7

**Published:** 2025-01-24

**Authors:** Lilla Nóra Kovács, Natália Kocsel, Zsófia Tóth, Tamás Smahajcsik-Szabó, Szilvia Karsai, Gyöngyi Kökönyei

**Affiliations:** 1 Institute of Psychology, ELTE Eötvös Loránd University, Budapest, Hungary; 2 Doctoral School of Psychology, ELTE Eötvös Loránd University, Budapest, Hungary; 3 NAP3.0-SE Neuropsychopharmacology Research Group, Hungarian Brain Research Program, Semmelweis University, Budapest, Hungary; 4 Faculty of Pharmaceutical Sciences, Department of Pharmacodynamics, Semmelweis University, Budapest, Hungary

**Keywords:** Human behaviour, Risk factors

## Abstract

Emotion regulation plays a crucial role in responding to daily stressors. While intrapersonal emotion regulation has received significant attention, the role of interpersonal emotion regulation remains understudied. This study aimed to explore the associations between intrapersonal perseverative cognition (i.e., rumination and worry) and interpersonal perseverative cognition (i.e., co-rumination), in the context of daily negative events. A daily diary study was conducted with a sample of university students (N = 178). The study comprised a baseline survey assessing trait-level variables, followed by a diary study where participants were sent an approximately two-minute-long survey every evening for 10 consecutive days. The findings indicated that daily co-rumination was associated with daily intrapersonal perseverative cognition, even when controlling for trait-level worry. The association between intra- and interpersonal perseverative cognition was stronger in the presence of daily negative events. Our findings indicate a need for further investigation into the contemporaneous relationship between co-rumination and intrapersonal rumination/worry, to ascertain their unique or joint adverse effects on mental health, especially within the context of daily negative events.

## Introduction

When we encounter stressors in our daily lives, it is crucial to effectively respond to the emotions they trigger. This process, known as emotion regulation (ER)^1^, has primarily been studied as an intrapersonal phenomenon. However, researchers increasingly recognize the significance of interpersonal ER as well^2^. Despite the importance of ER in our daily life, the association between intra- and interpersonal ER is understudied. For example, both rumination^3,4^ and its interpersonal variant, i.e. co-rumination^5^, have been subject of numerous studies, however they were rarely investigated together, particularly in an ecologically valid design. For this reason, in the current study, we aim to highlight the associations of rumination and its interpersonal manifestation, co-rumination in daily life, in the context of trait and state worry, and daily negative events.

Rumination is a repetitive and passive focus on the symptoms of distress, and the causes and consequences of these symptoms, without engaging in active problem-solving^6^. If it is employed as a stable response to distress or negative emotion, it is considered a maladaptive ER strategy. Consequently, trait rumination is a risk factor for psychopathology^7^. The interpersonal manifestation of rumination is co-rumination, in which dyads (e.g., friends, romantic partners, or mother–child pairs) repeatedly discuss their personal problems, associated symptoms, and possible underlying causes, consequences, mutually encouraging each other to further discuss these issues, while they focus on negative emotions^8^.

Worry is defined as a repetitive, future-oriented thought process that is often focused on potential negative outcomes or uncertainties, typically in the context of anxiety^9^. The research on rumination and worry has its roots in different theoretical frameworks. Worry was studied within the context of generalized anxiety disorder, while rumination was examined in relation to major depressive disorder^6,9^. Reflecting these independent origins, rumination and worry were measured separately using distinct instruments, until approximately the early 2000s. Subsequently, numerous empirical studies have demonstrated that these two constructs are distinct^10,11^ and should not be treated as interchangeable. Nevertheless, as with the overlapping characteristics of depressive and anxiety disorders, rumination and worry share several similar features, which lends support their joint analysis under the broader concept of perseverative thoughts^12^. Rumination and worry are similar in that both processes are repetitive, intrusive, negative, predominantly self-focused, difficult to control, and lead to abstract information processing^13^. Furthermore, both are considered as transdiagnostic characteristics, indicating their relevance across a range of disorders. Although worry was previously associated exclusively with generalized anxiety disorder, and rumination with depression, it is now understood that both repetitive thought patterns play a significant role in these disorders and contribute to the development and maintenance of a wide range of psychopathological symptoms. However, perseverative cognition can also be observed in the thought processes of healthy individuals^12,14^.

Rumination and co-rumination are related constructs since they are both characterized with a negative, repetitive, passive dwelling on problems^8^. Additionally, they were found to be positively associated both concurrently and prospectively^15^ and showed significant associations with internalizing problems^16^. However, co-rumination also involves self-disclosure, therefore, it can be associated with both positive (i.e., social support, increased emotional closeness and intimacy) and negative (i.e., internalizing symptoms, anxiety, depression) mental health outcomes^16–18^.

The phenomenon of co-rumination can be conceptualized as a form of socio-affective support, whereby mutual emotional stimulation facilitates the perception of shared emotional states between partners, thereby fostering an emotional communion and social integration^19^. These feelings can readily render the ruminative response by partners to the disclosure of personal problems and related emotions socially rewarding for both the disclosers and the respondents. Nevertheless, findings from laboratory studies indicate that socio-affective support during emotional disclosure without offering new interpretations will not mitigate the emotional impact of negative events^20^. Consequently, if the discourse remains focused exclusively on negative emotions and problems, without offering constructive interpretations or new meanings, it may not facilitate emotional recovery^19^, contributing to the negative mental health correlates of co-rumination. Moreover, a recent study revealed that individuals who engaged in co-rumination (as opposed to conversing naturally) about their most stressful extradyadic issue with their partner exhibited elevated physiological responses^21^.

As rumination prolongs the temporal extent of a stressor beyond the conventional period of reactivity^22^, it seems reasonable to investigate the extent to which intrapersonal and interpersonal versions of it coexist in everyday functioning. Similarly, in the case of worry, thoughts revolve around potential future events, which may or may not ever occur. Therefore, perseverative thought processes (i.e., rumination and worry) not only sustain but also actively generate stress, impacting both psychological and physiological domains^23^. Based on these observations, perseverative thoughts can be considered both stressors in themselves and mediators of stressor impacts^24^. Furthermore, previous research has shown that stress can increase emotional sharing, which, when coupled with a ruminative tendency at the individual level, is more likely to escalate into co-rumination^25,26^. Nonetheless, it remains unclear whether co-rumination subsequently increases intrapersonal rumination^27^ or, conversely, whether heightened intrapersonal rumination leads to more co-rumination^15^.

In studies employing ecological momentary assessments, such as in daily diary studies, individuals can report their intra- and/or interpersonal emotion regulation strategies over several days, thereby enabling the investigation of the association between them at the within-person level^4^. Diary studies also enable researchers to examine emotion regulation in relation to context. For example, a diary study found that ruminating in response to daily negative events moderated the relationship between the occurrence of such events and negative mood. On days when participants responded to daily hassles with rumination, these events were associated with negative mood; however, on days when participants did not ruminate on them, there was no association between stressful events and negative mood^28^. Negative events are known to easily trigger rumination, especially if the event highlights a discrepancy between the current state and a goal’s desired state^29^. Daily negative events can readily precipitate not only ruminative thoughts, but also worrisome ones^30^. Worry, defined as repetitive thoughts related to potential future threats^31^, is similar to rumination in that it is experienced as unpleasant and repetitive^32^. Furthermore, the circumstances in which these thoughts arise are perceived as beyond the participants’ control^32^. In light of these characteristics of perseverative cognitions, it seems reasonable to posit that those who ruminate or worry about something may engage in the sharing of their emotions and thoughts with others. Individuals may respond to shared emotions and thoughts in a number of ways^33^, including co-rumination. Subsequently, co-rumination may prolong intrapersonal rumination, as evidenced by a study that found that co-rumination was related to increased intrapersonal rumination the following day, while the reverse was not found^34^.

In the current study, we aimed to examine the concurrent relationship between intrapersonal perseverative cognition (i.e., rumination and worry) and interpersonal perseverative cognition (i.e., co-rumination) using a daily diary study, as literature on their relationship is scarce, especially using intensive longitudinal designs. More specifically, we hypothesized that daily co-rumination is associated with daily intrapersonal perseverative cognition (i.e., rumination and worry), even when controlling for trait-level intrapersonal perseverative cognition. Furthermore, we assumed that the relationship between daily co-rumination and daily intrapersonal perseverative cognition is moderated by whether anything negative occurred that day (in which case we hypothesized a stronger relationship).

This study was conducted during the COVID-19 pandemic, a period marked by heightened uncertainty, increased stress, and significant disruptions to daily life. The pandemic context likely intensified experiences of daily negative events and may have amplified the relevance of both intrapersonal and interpersonal emotion regulation strategies. Given the heightened stress and social isolation many individuals faced, the potential for perseverative cognition—including both rumination and co-rumination—may have been elevated. This context underscores the importance of examining how individuals manage daily stressors through both intra- and interpersonal mechanisms, as these insights could inform mental health interventions for future crisis situations.

## Methods

### Sample & procedure

We carried out a daily diary study with university students between June 2020 and May 2021 in four waves that comprised a baseline survey assessing trait-level variables, followed by a 10-day-long diary study where participants were sent an approximately 2-min-long survey every morning and evening. Each survey was conducted in Qualtrics. Some of the findings from this data collection have been previously reported with focusing on a different question: the relationship between daily negative rumination, positive rumination and their relationship with negative and positive affect were published elsewhere^35^.

In the present analysis, only the evening surveys were included in the current investigation since the questions related to our hypotheses were asked in the evening. Participants were emailed the links to the evening surveys at 8 p.m. and were encouraged to complete them right before bedtime, but no later than 2 a.m. Following the diary study, participants were given a final questionnaire in which they were asked to provide comments on the study and whether the recurring surveys changed their week or mood. Students were recruited via university courses and were given partial class credit in exchange. Every participant provided written informed consent and participated voluntarily and anonymously. 189 students consented to participate; however, 8 were eliminated owing to low compliance (< 50%) and one for failing to complete the baseline survey. Although students with a history of mental or neurological diseases (n = 1) or currently undergoing intensive psychotherapy (n = 1) were permitted to participate, their data was not analyzed. 178 participants could be included in the final sample (158 females; mean age = 22.72, SD = 3.89 years). Kleinman Lab’s online calculator ‘Power Curves for Multi-level Studies’ (The State University of New Jersey, https://kleimanlab.org/resources/power-curves/) was used to conduct a power analysis. Medium to large effects (d = 0.5–0.8) were expected between daily co-rumination and daily perseverative cognition based on a recent meta-analysis on emotion regulation strategies measured with intensive longitudinal studies^4^. The estimated power (1 − β) for our sample of 178 participants with 10 time points and > 50% compliance is > 0.9. Ethical consent was obtained from the Institutional Review Board (approval number: 2020/203). Data collection was in accordance with the Declaration of Helsinki.

### Measures

*Trait-level depressive rumination* was assessed using the Ruminative Response Scale short form^36^. This instrument consists of 10 items that cover two facets of ruminative thinking: brooding (passively dwelling on sad mood, its causes and consequences) and reflective pondering (a more purposeful response aimed at analyzing emotions or events in order to understand them). A 4-point Likert scale (ranging from 1 = “never” to 4 = “always”) was employed to measure the extent to which the given statements characterized the respondents when they are in a low mood. The internal consistency for the total score of the scale in the current study was good (Cronbach’s alpha was 0.70), similar to previous studies using the Hungarian adaptation of the scale^37,38^.

*Trait-level worry* was measured using the ultra-brief version of the Penn State Worry Questionnaire (PSWQ)^39^. The original 16-item instrument^40^ was condensed to three items, with participants rating the items on a Likert scale ranging from 1 = "not at all typical of me" to 5 = "very typical of me". The psychometric properties of this ultra-brief version in Hungarian have been shown to be good^41^, as they were in the present study (Cronbach’s alpha was 0.89).

*Daily co-rumination* was measured using four items of the Co-rumination Questionnaire^8^. These questions were only assessed if participants answered “yes” to the question “Did you have a problem today that you discussed with someone close to you?”, to which we refer to as “daily problem-talk”. A subset of questions was modified to capture daily co-rumination behaviors instead of trait-like co-rumination: “When we saw each other today, we talked about this problem even if we had planned to do something else together.”; “We talked about every part of the problem over and over.”; “We tried to figure out every one of the bad things that might happen because of the problem.”; “We talked a lot about how bad I felt because of the problem.” Participants rated their responses on a 5-point Likert scale, ranging from 1 = “never” to 5 = “almost always”. Items demonstrated adequate internal consistency: within-person omega was 0.69 [0.63–0.74], and between-person omega was 0.84 [0.78–0.91].

*Daily perseverative cognition (rumination and worry)* was assessed with two items, as recommended by Moberly and Watkins^42^ and Kircanski and colleagues^43^. The items were as follows: “Today, I was dwelling on my feelings and problems”; “Today I was worried about things that could happen”. Participants were asked to rate their responses to both items on a 7-point Likert scale, ranging from 1 = “never” to 7 = “almost always”. The sum of the two items was used as daily perseverative cognition score. Items demonstrated good internal consistency: within-person omega was 0.74 [0.71–0.77], and between-person omega was 0.92 [0.89–0.96].

*Daily negative events* were measured by asking participants to report whether a negative or unpleasant event occurred on that day (dichotomous, yes or no answers were collected).

### Statistical analyses

Data was analyzed with multilevel regression in R using the *esmpack*^44^ and the *nlme* packages^45^. Data and scripts are available at https://osf.io/rnj7p/. We estimated intraclass correlations of the time-variant variables and utilized linear mixed-effects models with constrained maximum loglikelihood estimation for hypothesis testing. Daily co-rumination was the outcome measure, and the predictors were daily perseverative cognition and daily negative events. We also tested the interaction of daily negative events and daily perseverative cognition. To distinguish between- and within-person variance, between-person (i.e., the mean score of the item for every individual) and within-person mean centered (i.e., the difference between the participant’s own mean score and their specific response at the given timepoint) values for daily perseverative cognition were entered separately in the model. We tested the association between co-rumination and the following potential confounding factors: trait-level rumination, trait-level worry, gender, age, and whether the study affected participants’ mood or week in a separate multilevel regression model. Among these, only trait worry was significantly associated with co-rumination, therefore, we controlled for trait worry in the model. Although not significant, we also controlled for gender, due to the uneven gender distribution of our sample.

## Results

The descriptive statistics of the assessed continuous variables are demonstrated in Table 1. Mean compliance rate was 90.39% (SD = 13.71), with a minimum of 50% and a maximum of 100%.

**Table 1:** Descriptive statistics of the assessed continuous variables (N = 178).

	Minimum	Maximum	Mean	SD
Trait rumination	14	35	24.27	4.46
Trait worry	3	15	8.26	3.26
Daily co-rumination	4	20	–	–
Daily perseverative cognition	2	14	–	–

**Fig. 1:**
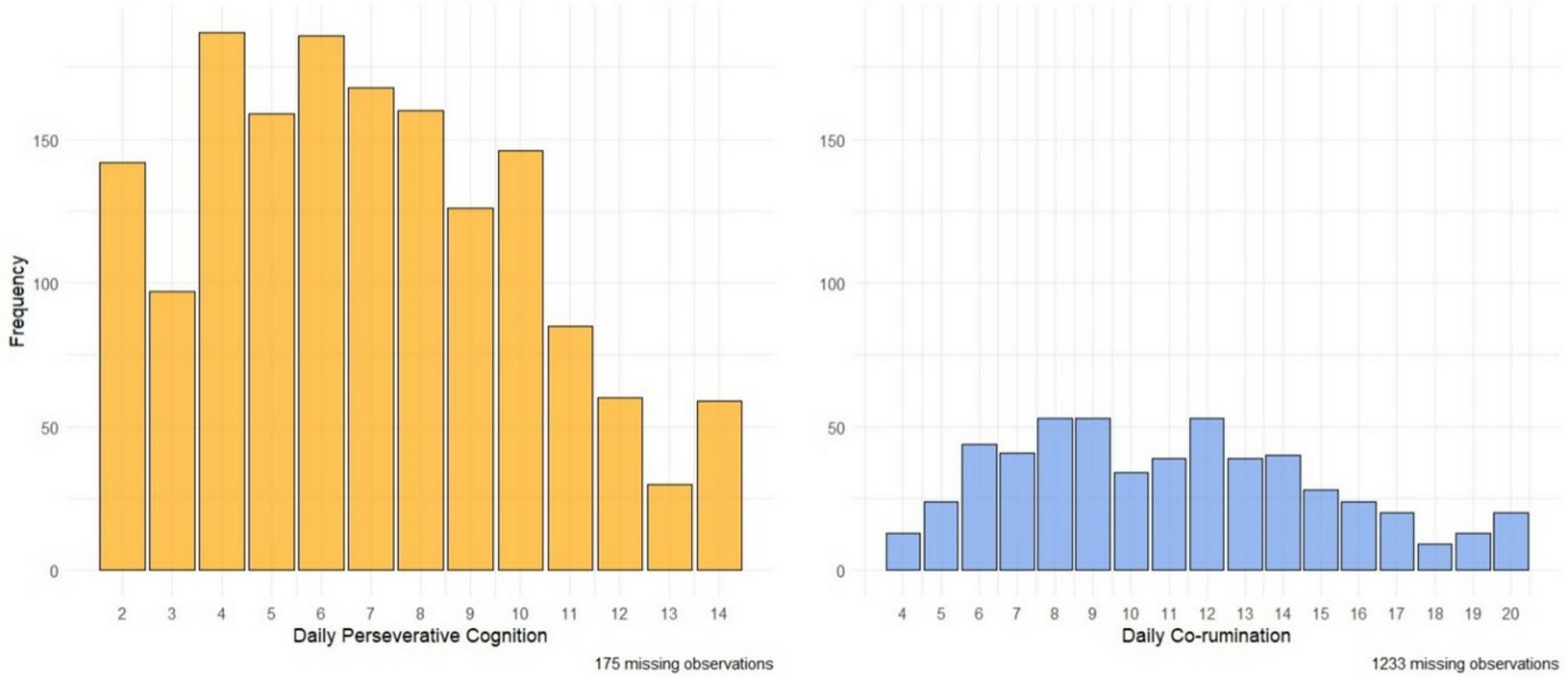
Overall frequency distributions of time-varying measures during the 10-day sampling period. N = 178. Number of observations = 1605/547.

As for the categorical variables, participants reported that a negative event occurred on that day 436 times (27.2%). Participants engaged in daily problem-talk 547 times out of 1605 timepoints (34.1%), and of these instances, participants reported that a negative event occurred on that day 229 times. Due to the amount of missing data, power analysis was recalculated. However, mean compliance was much higher than the initially estimated 50% (90.39%). Therefore, for a mean completion rate of approximately 30%, the estimated power (1 − β) is 0.8 for medium to large effects (d = 0.5–0.8). The frequencies of scores of the time-varying variables across all observations are demonstrated in Fig. 1.

**Fig. 2:**
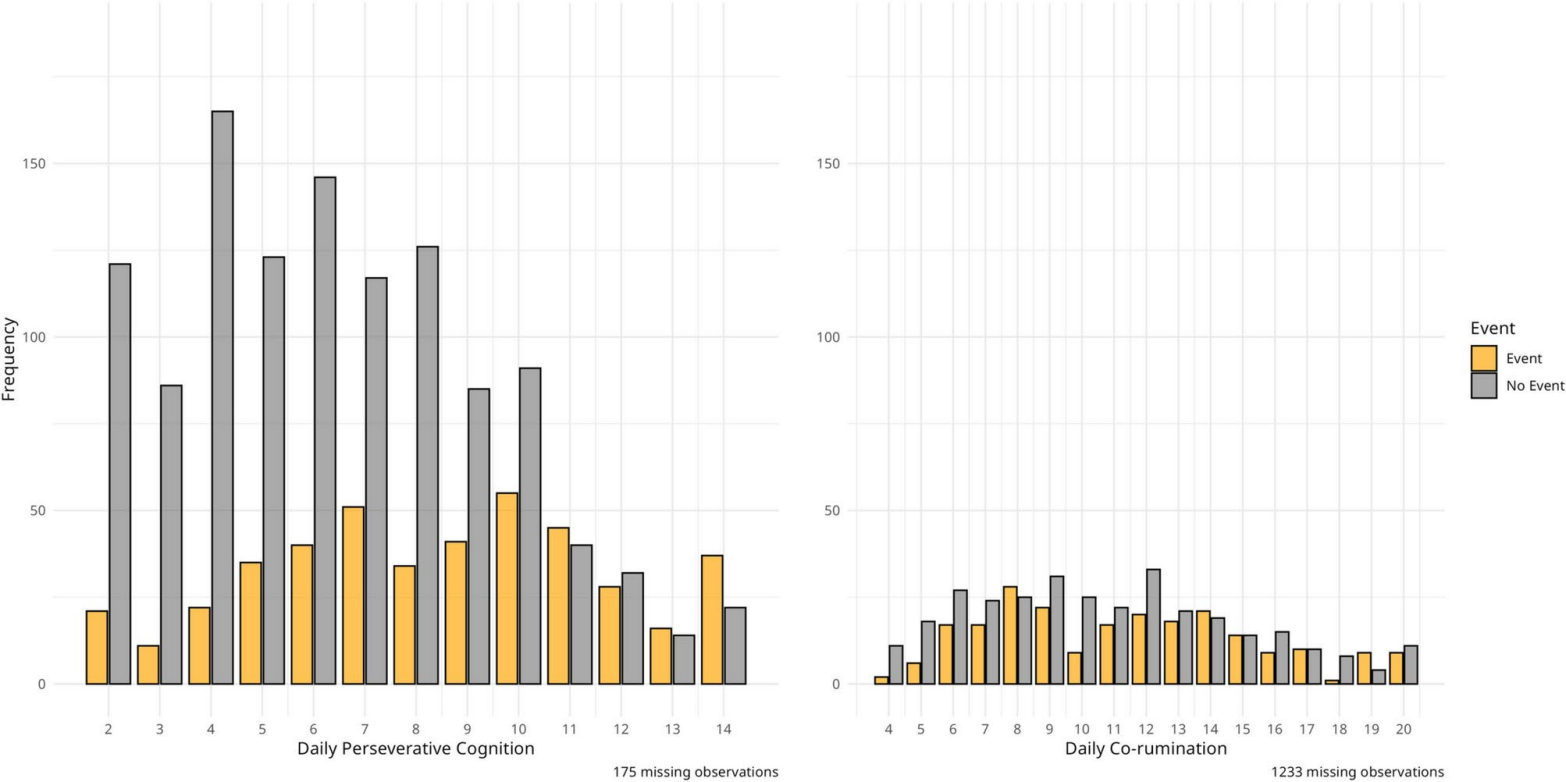
The frequency of daily perseverative cognition and daily co-rumination scores on days with and without a negative event. N = 178. Number of observations = 1605/547. Event = A negative event occurred on that day; No event = A negative event did not occur that day.

Figure 2 demonstrates the frequency scores of daily perseverative cognition and daily co-rumination grouped by whether a negative event occurred on that day or not. The figure shows that on days without a negative event, lower daily perseverative cognition and daily co-rumination scores are more frequently reported, but people appear to engage in both processes on days without negative events as well.

Table 2 presents the correlations between key study variables, including both within-person (daily variations) and between-person (trait-level) measures of co-rumination and perseverative cognition. Including both levels of correlation allows us to capture the consistency and variability of these relationships across individuals as well as within each individual’s daily experiences. The within-person correlation between daily co-rumination and daily perseverative cognition (r = 0.39, p ≤ 0.05) highlights that on days when participants engage in more co-rumination, they also tend to experience increased intrapersonal perseverative cognition. The between-person correlation (r = 0.20, p ≤ 0.05) indicates that individuals with higher tendencies to worry or ruminate within the sampling period also report somewhat higher daily levels of co-rumination.

**Table 2:** Pearson correlations of the assessed daily person-level and trait-level continuous variables.

	Trait worry	Daily co-rumination	Daily perseverative cognition
Trait-level			
Trait rumination	0.49*	0.19*	0.22*
Trait worry	–	0.21*	0.28*
Daily co-rumination	–	–	0.31*
Between-person			
Daily co-rumination	–	–	–
Daily perseverative cognition	–	0.20*	–
Within-person			
Daily co-rumination	–	–	
Daily perseverative cognition	–	0.39*	

N = 178. *p ≤ 0.01.

Intraclass correlations (ICC) were 0.43 for daily co-rumination, and 0.35 for daily perseverative cognition. First the association of potential confounds with co-rumination were tested (see Table S1 of the Supplementary Material), among which only trait worry was significantly associated with co-rumination, therefore, trait worry was controlled for in the model. Furthermore, although not significant, we included gender due to the uneven gender distribution of our sample.

Next, we conducted a linear mixed-effects model with daily co-rumination as the outcome measure using restricted maximum likelihood estimation, as shown in Table 3. The model included random intercepts to account for inter-individual variability in baseline levels of co-rumination, recognizing that each participant may have a unique starting level of co-rumination. Random slopes were included for daily perseverative cognition to allow for individual differences in its relationship with daily co-rumination.

**Table 3:** Linear mixed-effects model with restricted maximum log likelihood estimation.

	B	SE	df	t	p	var	ρ01
Intercept	7.510	2.510	375	2.992	0.003		
Gender	− 1.200	0.835	164	− 1.437	0.153		
**Trait worry**	**0.162**	**0.078**	**164**	**2.089**	**0.038**		
**Daily perseverative cognition plm**	**0.567**	**0.259**	**164**	**2.191**	**0.030**		
**Daily perseverative cognition wpmc**	**0.632**	**0.199**	**375**	**3.175**	**0.002**		
Daily negative event	0.203	1.106	375	0.183	0.855		
Daily negative event * daily perseverative cognition plm	− 0.031	0.144	375	− 0.218	0.828		
Daily negative event * daily perseverative cognition wpmc	**− 0.243**	**0.121**	**375**	**− 2.001**	**0.046**	0.053	-0.38
*Marginal R*^2^*/conditional R*^2^	*0.139/0.503*						
Residual variance (σ^2^)	8.485						

Dependent variable: daily co-rumination.

N = 178. Number of observations = 547. Wpmc = within-person mean centered; plm = person-level mean; Marginal R^2^ = variance explained by fixed effects; conditional R^2^ = variance explained by fixed and random effects; var = variance of random slopes; ρ01 = correlation of random slope with random intercept. Results in bold are significant.

Trait worry showed a significant positive association with daily co-rumination (B = 0.162, SE = 0.078, p = 0.038), suggesting that individuals with higher trait worry tended to co-ruminate more frequently. Daily perseverative cognition, both at the between-person level (B = 0.567, SE = 0.259, p = 0.030) and within-person level (B = 0.632, SE = 0.199, p = 0.002), was significantly associated with increased daily co-rumination. Daily negative events did not significantly predict co-rumination (B = 0.203, SE = 1.106, p = 0.855). Additionally, the interaction between daily negative events and between-person daily perseverative cognition was not significant (B = − 0.031, SE = 0.144, p = 0.828), indicating that individuals who generally engage in higher levels of daily perseverative cognition do not necessarily experience increased daily co-rumination on days with more negative events. However, there was a significant interaction between daily negative events and daily within-person perseverative cognition (B = − 0.243, SE = 0.121, p = 0.046), where, on days with a negative event, the relationship between co-rumination and within-person perseverative cognition was stronger. The model’s marginal R^2^ (variance explained by fixed effects) was 0.139, while the conditional R^2^ (variance explained by both fixed and random effects) was 0.503.

**Fig. 3:**
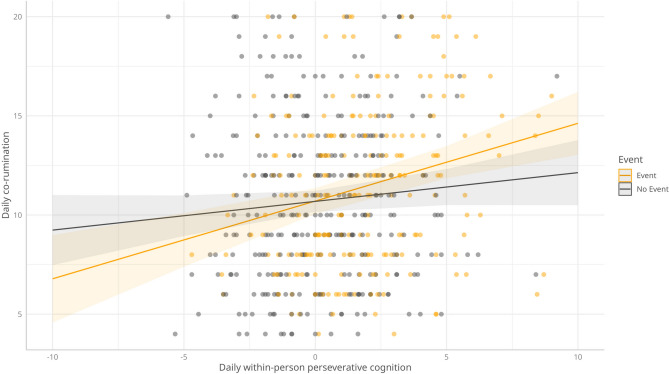
The interaction between daily negative events and within-person daily perseverative cognition in relation to daily co-rumination. N = 178. Number of observations = 547. Event = A negative event occurred on that day; No event = A negative event did not occur that day.

The interactions between daily negative events and (within-person) daily perseverative cognition is visualized in Fig. 3.

### Sensitivity analyses

Given that between-person mean-centered daily perseverative cognition and trait worry/rumination also reflect individual differences, one might argue that the between-person mean variable is the sole necessary construct to account for these differences. However, in consideration of the extant literature^42,46^, there is evidence that individuals with higher levels of trait worry and rumination are more likely to experience perseverative thoughts in their daily lives. It is also important to note that state-level rumination and worry may differ significantly from trait-level, personality-based characteristics. During periods of elevated stress, individuals may experience an increase in perseverative thoughts. Consequently, the mean level of perseverative cognition over a few days may not be an accurate reflection of the individual’s overall trait-level cognitive style. To address this issue, a sensitivity analysis was conducted by rerunning the model without trait worry, which yielded identical results (see Table S2 of the Supplementary Material).

The results of experience sampling method studies^47,48^ indicate that intense negative emotions can effectively trigger rumination (along with other emotion regulation strategies). Similarly, negative events are reasonably assumed to elicit negative emotions^49^. It can thus be assumed that on days when negative emotions are heightened due to stressful (negative) events, individuals may choose strategies, such as emotional sharing or co-rumination, that are believed to mitigate negative affect. Consequently, the model was recalibrated to include daily negative affect and its interaction with negative events (see Table S3 of the Supplementary Material for details). However, neither of these variables emerged as statistically significant.

## Discussion

Co-rumination is typically studied in the context of friendship and its relation to internalizing symptoms; however, knowledge on how co-rumination is related to intrapersonal rumination remains limited^5^. Moreover, studies about co-rumination primarily focus on teenagers, while studies involving adults are scarce. Hence, this study aimed to address these gaps by exploring the relationship between intrapersonal perseverative cognition on problems, emotions, or future events and daily ruminative problem discussions with others among young adults. Generally, this study found a significant positive linear association between daily within-and between-person perseverative cognition and daily co-rumination. In other words, participants who experienced more perseverative thoughts over the course of 10 days were more inclined to engage in an excessive amount of problem talk with others. Furthermore, participants reported engaging in more ruminative problem discussions with others on days when their intrapersonal rumination exceeded their personal average. These findings are in line with the proposal of Rose^8^, i.e., that co-rumination may be related to internalizing symptoms via exacerbating intrapersonal rumination.

Furthermore, our results indicated that while daily negative events were not directly associated with daily co-rumination, the occurrence of a negative event on the given day moderated the association between daily co-rumination and perseverative cognition. In other words, on days when participants reported a negative event, the relationship between intra-and interpersonal rumination was stronger. This indicates that negative events appear to facilitate the association between intra-and-interpersonal rumination/perseverative cognition, which could amplify the maladaptive aspect of co-rumination/problem-talk. It may initially seem surprising that, even on non-stressful days, levels of both intra- and interpersonal emotion regulation remained relatively high. One might expect that, in the absence of distinctly negative events, individuals would have little need for emotion regulation. However, perseverative cognition may also be triggered by past events or future worries even in the absence of current negative events, especially among people with strong trait-level tendencies to engage in rumination and/or worry^46^. Numerous studies indicate that emotion-triggering events (both positive and negative) facilitate social sharing^33^, however, there are other ways to respond to these stories besides engaging in co-rumination, as also shown by the frequency of co-rumination scores in our sample. In the framework of interpersonal emotion regulation, we can differentiate intrinsic and extrinsic strategies, where intrinsic strategies aim to modulate one’s own affect, and the extrinsic strategies aim to alter the other person’s affect; furthermore, these strategies could be response-independent or response-dependent, where the former does not require a specific response from the other person in order to change one’s affect, while the latter does^2^. For instance, when someone shares a negative event, the other person could respond with supportive behavior, empathy or altruism, which are response-dependent strategies that generally improve the sharer’s mood via demonstrating affiliation^2^.

Strengths of this study include stronger ecological validity compared to cross-sectional self-report data, and the study of co-rumination, an understudied form of interpersonal emotion regulation, and its relationship with both intrapersonal (i.e., daily perseverative cognition) and contextual (i.e., daily stressful events) factors. While it might seem logical to assume a relationship between intrapersonal rumination/worry and interpersonal co-rumination, this issue has rarely been explored in research directly. Furthermore, models addressing intrapersonal rumination^6^ consistently emphasize that ruminative individuals tend to display cognitive and behavioral passivity. This passivity is, in part, attributed to depressive symptoms that often co-occur with rumination, as well as to the social friction or isolation that can emerge as a result of persistent ruminative thought patterns^50,51^. Nevertheless, some studies have indicated that individuals who engage in rumination may seek social support more frequently during periods of stress than those who do not ruminate. However, they are often less satisfied with the support they receive^52^. In light of the aforementioned findings, our manuscript presents a particularly compelling result: a high frequency of individual perseverative thoughts is associated not with withdrawal, but rather with interpersonal co-rumination, which can also be conceptualized as a form of seeking and providing social support. Consequently, co-rumination may serve as a source of socio-affective support for individuals engaged in rumination (Rimé, 2020), particularly on days marked by negative events, when they tend to ruminate more than they typically do.

There are constructs that may exhibit similarities to co-rumination. For example, venting also involves self-disclosure and an excessive focus on expressing one’s emotions, however, venting prioritizes emotional relief over listener response^53^. Furthermore, recent conceptualizations differentiate co-rumination from verbal rumination, which is defined as the act of repeatedly disclosing personal problems or negative emotions to a close other^54^, yet the other individual does not encourage the discloser to dwell on the problems. It is also referred to as social rumination^55^. The consequences of verbal rumination are partially dependent on the quality of support provided by the listener. For instance, satisfaction with friendship is higher among those who receive good support when engaging in verbal rumination. However, anxiety and intrapersonal brooding after verbal rumination were high regardless of whether poor or good support was received^54^.

The motivation behind the disclosure of personal issues and related emotions in a social context may vary considerably^56^. One common motive is seeking comfort or consolation^56^. However, excessive reassurance seeking is considered to be a maladaptive interpersonal emotion regulation strategy whereby the individual repeatedly keeps asking for assurance from others^53^. Though some findings indicate that excessive reassurance seeking is positively correlated with co-rumination^57^, more research is needed to understand how different interpersonal emotion regulation strategies relate to each other. Although self-disclosure or co-rumination is frequently driven by the desire to alleviate negative emotions, their occurrence in social settings highlights the importance of examining how social goals or motivations^58^ influence the positive and negative correlates of co-rumination. Moreover, future research would benefit from a closer examination of the specific contents of co-ruminative episodes. For example, DiGiovanni and colleagues^55^ posit that co-ruminating on significant societal issues and experiencing heightened negative affect during the co-ruminative episode may result in collective action.

Our study has several drawbacks. First, our sample comprised of university students, therefore results are less generalizable. On the other hand, university students are vulnerable to psychological problems, therefore investigating their mental wellbeing is of high importance^59,60^. Moreover, given that previous studies^61^ have predominantly focused on examining co-rumination within adolescent samples, there is a need for future research among older individuals. Another drawback is our sample’s unequal gender distribution, with a disproportionate number of female participants. Furthermore, information on the history of psychiatric and/or neurological diseases was collected through a self-reported item rather than a diagnostic interview, which may affect the accuracy of these rates. Moreover, the present analyses do not allow us to draw causal conclusions. We could not perform lagged analyses, because we only measured the daily level of co-rumination among participants who reported that they discussed a problem with someone on that given day, resulting in a lot of missing values. Furthermore, we did not assess the personal advantages (e.g., feeling close to others) and disadvantages (e.g., elevated levels of anxiety and depression) of co-rumination that could highly vary from person to person^62^. Furthermore, our items measuring co-rumination did not account for online forms of co-rumination, which may be an increasingly relevant aspect of social exchanges^63^. Expanding the study of co-rumination to digital contexts and examining potential differences between in-person and online co-rumination could be a valuable direction for future research. Moreover, the items we used to assess perseverative cognition do not exclude the possibility of interpersonal processes, which might obscure results. Future studies that compare intra-and interpersonal perseverative cognition should reconsider item phrasing to better distinguish intra-and interpersonal perseverative cognition. Another limitation is that we did not assess the type or intensity of daily negative events, which could also be an important factor in whether they trigger co-rumination or intrapersonal perseverative cognition. For instance, interpersonal stressors tend to trigger more co-rumination (and rumination), as due to their ambiguity they require more effort to interpret^5^. Future studies should also consider the type and intensity of daily negative events and their different roles in triggering co-rumination and interpersonal emotion regulation in general.

It is important to note that trait worry, rather than trait rumination, was significantly associated with daily co-rumination. Individuals who experience high levels of worry may frequently engage in interpersonal emotion regulation strategies to regulate their negative emotions by involving others, of which co-rumination could be one of several strategies, also referred to as anxiety talk^64^. To gain a better understanding of this relationship, additional research is required on the link between worry and intrinsic interpersonal emotion regulation.

In conclusion, this study delved into the relationship between co-rumination and intrapersonal perseverative cognition among young adults, shedding light on an area that has been understudied, particularly in adult populations. The implications of these findings underscore the importance of understanding the interplay between intrapersonal rumination and interpersonal communication, particularly in the context of mental health interventions. Recognizing the potential associations between co-rumination and intrapersonal perseverative cognition highlights the need for interventions that promote healthier forms of social support and emotion regulation strategies, ultimately contributing to improved mental well-being among young adults.

## Supplementary Information

Supplementary Tables.

## Data Availability

Data and scripts are available at https://osf.io/rnj7p/.
