# Supplementary material for: The daily relations of co-rumination and perseverative cognition
Source: Sci Rep. 2025 Jan 24;15:3021. doi: 10.1038/s41598-025-87335-7 (PMC11757728; doi:10.1038/s41598-025-87335-7)
Supplement: Supplementary file 1 — Supplementary Tables. [file 41598_2025_87335_MOESM1_ESM.pdf]

Supplementary Material

The daily relations of co-rumination and perseverative cognition

Lilla Nóra Kovács[1], Natália Kocsel[1], Zsófia Tóth[1,2], Tamás Smahajcsik-Szabó[1,2], Szilvia Karsai[1,2], Gyöngyi Kökönyei[1,3,4,*]

[1]Institute of Psychology, ELTE Eötvös Loránd University, Budapest, Hungary

[2]Doctoral School of Psychology, ELTE Eötvös Loránd University, Budapest, Hungary

[3]NAP3.0-SE Neuropsychopharmacology Research Group, Hungarian Brain Research Program, Semmelweis University, Budapest, Hungary

[4]Faculty of Pharmaceutical Sciences, Department of Pharmacodynamics, Semmelweis University, Budapest, Hungary

[*]Corresponding author at kokonyei.gyongyi@ppk.elte.hu.

Table S1.

Linear model of potential confounds with restricted maximum log likelihood estimation.

Dependent variable: daily co-rumination

	B	SE	df	t	p
Intercept	9.005	2.697	375	3.339	<0.001
age	-0.058	0.065	159	-0.893	0.373
gender	-0.504	0.873	159	-0.577	0.565
study affected my week	0.108	0.208	159	0.519	0.604
study affected my mood	-0.097	0.185	159	-0.525	0.600
trait rumination	0.105	0.063	159	1.681	0.095
**trait worry**	**0.203**	**0.088**	**159**	**2.300**	**0.023**
R^2			0.108		
Residual variance (σ^2)			9.368		

Notes. N = 178. Number of observations = 547. Wpmc = within-person mean centered; plm = person-level mean; Marginal R2 = variance explained by fixed effects; conditional R2 = variance explained by fixed and random effects; var = variance of random slopes; ρ01 = correlation of random slope with random intercept. Results in bold are significant.

Table S2.

Linear model with restricted maximum log likelihood estimation. Dependent variable: daily co-rumination

	B	SE	df	t	p	var	ρ01
Intercept	8.10	1.86	375	4.35	<0.01		
gender	-1.10	0.84	165	-1.31	0.19		
**Daily perseverative cognition plm**	**0.65**	**0.14**	**165**	**4.52**	**<0.01**		
**Daily perseverative cognition wpmc**	**0.39**	**0.09**	**375**	**4.25**	**<0.01**		
Daily negative event	0.20	1.11	375	0.18	0.86		
Daily negative event * daily perseverative cognition plm	-0.03	0.14	375	-0.23	0.82		
**Daily negative event * daily perseverative cognition wpmc**	**-0.24**	**0.12**	**375**	**-1.99**	**0.04**	-0.36	
Marginal R^2/conditional R^2	0.127/0.499						
Residual variance (σ^2)	8.52						

Notes. N = 178. Number of observations = 547. Wpmc = within-person mean centered; plm = person-level mean; Marginal R^2 = variance explained by fixed effects; conditional R^2 = variance explained by fixed and random effects; var = variance of random slopes; ρ01 = correlation of random slope with random intercept. Results in bold are significant.

Table S3.

Linear model of daily negative affect and the interaction between negative affect and negative event with restricted maximum log likelihood estimation. Dependent variable: daily co-rumination

	B	SE	df	t	p	var	ρ01
Intercept	7.97	1.89	371	4.22	<0.01		
gender	-1.15	0.84	164	-1.38	0.17		
**Daily perseverative cognition plm**	**0.56**	**0.19**	**164**	**2.96**	**<0.01**		
**Daily perseverative cognition wpmc**	**0.38**	**0.11**	**371**	**3.57**	**<0.01**		
Daily negative event	0.22	1.16	371	0.19	0.85		
Daily negative affect plm	0.33	0.47	164	0.70	0.49		
Daily negative affect wpmc	0.09	0.23	371	0.40	0.69		
Daily negative event * daily perseverative cognition plm	-0.03	0.14	375	-0.23	0.82		
Daily negative event * daily perseverative cognition wpmc	-0.22	0.14	371	-1.61	0.10		

Daily negative event * daily negative affect plm	-0.18	0.50	371	-0.36	0.72		
Daily negative event * daily negative affect wpmc	-0.21	0.32	371	-0.65	0.52	0.053	-0.38
Marginal R^2/conditional R^2		0.13/0.52					
Residual variance (σ^2)		8.26					

Notes. N = 178. Number of observations = 547. Wpmc = within-person mean centered; plm = person-level mean; Marginal R2 = variance explained by fixed effects; conditional R2 = variance explained by fixed and random effects; var = variance of random slopes; ρ01 = correlation of random slope with random intercept. Results in bold are significant.

The daily negative affect items were selected based on the 10-item negative affect subscale of the Positive and Negative Affect Schedule (PANAS[1]), along with items used in the Experience-Sampling Study (ESM) conducted by Kircanski et al.[2]. The adjectives comprising the final set were selected from a broader list of items based on the findings of the pilot study. Participants were requested to indicate on a 7-point Likert scale, ranging from 1 (not at all) to 7 (very often), how often they felt sad, upset, angry, and irritable during the day. To create an aggregate measure of daily negative affect, the mean score of the four adjectives was calculated. The internal consistency of the items was found to be excellent, with within-person omega values of 0.81 [0-80-0.83], and between-person omega values of 0.91 [0.88–0.94]. For further details, see Kovacs et al., 2023[3].

References:

1 Watson, D., Clark, L. A. & Tellegen, A. Development and validation of brief measures of positive and negative affect - the PANAS Scales. *Journal of Personality and Social Psychology* **54**, 1063-1070 (1988). https://doi.org/10.1037/0022-3514.54.6.1063

2 Kircanski, K., Thompson, R. J., Sorenson, J., Sherdell, L. & Gotlib, I. H. The everyday dynamics of rumination and worry: precipitant events and affective consequences. *Cognition & Emotion* **32**, 1424-1436 (2018). https://doi.org/10.1080/02699931.2017.1278679

3 Kovács, L. N. *et al.* Associations between daily affective experiences, trait and daily rumination on negative and positive affect: a diary study. *J Pers* (2023). https://doi.org/10.1111/jopy.12897
